# Trial of an mHealth intervention to improve HIV prophylaxis for female sex workers, United Republic of Tanzania

**DOI:** 10.2471/BLT.24.291516

**Published:** 2024-10-29

**Authors:** Christopher H Mbotwa, Method R Kazaura, Kåre Moen, Christopher R Sudfeld, Emmy Metta, Melkizedeck T Leshabari, Muhammad Bakari, Elia J Mmbaga

**Affiliations:** a Department of Epidemiology and Biostatistics, Muhimbili University of Health and Allied Sciences, P.O. Box 65015, Dar es Salaam, 11103, United Republic of Tanzania.; b Department of Community Medicine and Global Health, University of Oslo, Oslo, Norway.; c Department of Global Health and Population, Harvard T. H. Chan School of Public Health, Boston, United States of America.; d Department of Behavioural Sciences, Muhimbili University of Health and Allied Sciences, Dar es Salaam, United Republic of Tanzania.; e Department of Internal Medicine, Muhimbili University of Health and Allied Sciences, Dar es Salaam, United Republic of Tanzania.

## Abstract

**Objective:**

To evaluate the effect of a mobile health (mHealth) intervention on early retention of female sex workers in human immunodeficiency virus (HIV) pre-exposure prophylaxis services in the United Republic of Tanzania.

**Methods:**

The study involved 783 female sex workers: 470 from Dar es Salaam who were given the *Jichunge* mHealth application (app) in addition to standard HIV pre-exposure prophylaxis (intervention arm), and 313 from Tanga who received pre-exposure prophylaxis alone (control arm). Participants were recruited using respondent-driven sampling and followed up for 12 months. Early retention was defined as attending a pre-exposure prophylaxis follow-up clinic within 28 days of an appointment scheduled for 1 month after starting treatment. To assess if the *Jichunge* app led to higher retention, we conducted intention-to-treat and per-protocol analyses using a regression model adjusted by inverse probability weighting.

**Findings:**

Early retention in HIV pre-exposure prophylaxis care was observed in 27.6% (130/470) of participants in the intervention arm and 20.1% (63/313) in the control arm. In the adjusted, intention-to-treat analysis, early retention was observed in 29.4% in the intervention arm and 17.7% in the control arm (risk difference: 11.8 percentage points; 95% confidence interval: 5.3–18.3).

**Conclusion:**

Early retention in HIV pre-exposure prophylaxis care was significantly greater among female sex workers in the United Republic of Tanzania who used the *Jichunge* app than in those who did not. Nevertheless, more than two thirds of sex workers using the application did not attend follow-up services after 1 month, suggesting that additional interventions are needed.

## Résumé

**Objectif:**

Évaluer l'impact d'une intervention de santé mobile (mHealth) sur l'adhésion précoce des travailleuses du sexe vis-à-vis des services de prophylaxie préexposition pour le virus de l'immunodéficience humaine (VIH) en République-Unie de Tanzanie.

**Méthodes:**

Cette étude a été menée auprès de 783 travailleuses du sexe: 470 d'entre elles étaient à Dar es Salaam et ont eu accès à l'application mHealth (app) *Jichunge* en complément d'une prophylaxie préexposition standard pour le VIH (volet d'intervention), tandis que les 313 autres provenaient de Tanga et n'ont bénéficié que d'une prophylaxie préexposition (volet de contrôle). Les participantes ont été sélectionnées par le biais d'un échantillonnage fondé sur les répondants, puis suivies pendant 12 mois. Une adhésion précoce signifiait assister à une consultation de suivi de la prophylaxie préexposition dans les 28 jours suivant un rendez-vous fixé un mois après le début du traitement. Enfin, pour déterminer si l'app *Jichunge* améliorait le taux d'adhésion, nous avons effectué des analyses selon l'intention de traiter et selon le respect du protocole, à l'aide d'un modèle de régression ajusté en fonction d'une pondération par l'inverse de la probabilité.

**Résultats:**

Nous avons observé une adhésion précoce au traitement de prophylaxie préexposition pour le VIH chez 27,6% (130/470) des participantes dans le volet d'intervention, contre 20,1% (63/313) dans le volet de contrôle. L'analyse ajustée selon l'intention de traiter a permis de constater une adhésion précoce de 29,4% dans le volet d'intervention et de 17,7% dans le volet de contrôle (différence de risque: 11,8 points de pourcentage; intervalle de confiance de 95%: 5,3-18,3).

**Conclusion:**

En République-Unie de Tanzanie, l'adhésion précoce au traitement de prophylaxie préexposition pour le VIH s'est révélée sensiblement plus élevée parmi les travailleuses du sexe ayant utilisé l'app *Jichunge* que parmi celles n'y ayant pas eu recours. Cependant, plus des deux tiers des travailleuses du sexe utilisant l'application ne se sont pas présentées aux services de suivi un mois plus tard, ce qui indique que d'autres mesures sont requises.

## Resumen

**Objetivo:**

Evaluar el efecto de una intervención de salud móvil (mHealth) sobre la retención temprana de las trabajadoras del sexo en los servicios de profilaxis previa a la exposición al virus de la inmunodeficiencia humana (VIH) en la República Unida de Tanzania.

**Métodos:**

En el estudio, participaron 783 trabajadoras del sexo: 470 de Dar es Salaam que recibieron la aplicación *Jichunge* mHealth además de la profilaxis estándar previa a la exposición al VIH (grupo de intervención) y 313 de Tanga que solo recibieron profilaxis previa a la exposición (grupo de control). Se seleccionó a los participantes mediante un muestreo dirigido por los propios encuestados y se les hizo un seguimiento durante 12 meses. La retención temprana se definió como la asistencia a una consulta de seguimiento de profilaxis previa a la exposición en los 28 días siguientes a una cita programada para 1 mes después de iniciar el tratamiento. Para evaluar si la aplicación *Jichunge* conducía a una mayor retención, se realizaron análisis por intención de tratar y por protocolo mediante un modelo de regresión ajustado por ponderación de probabilidad inversa.

**Resultados:**

El 27,6% (130/470) de los participantes del grupo de intervención y el 20,1% (63/313) del grupo de control mantuvieron la profilaxis previa a la exposición al VIH. En el análisis ajustado por intención de tratar, se observó una retención temprana del 29,4% en el grupo de intervención y del 17,7% en el grupo de control (diferencia de riesgo: 11,8 puntos porcentuales; intervalo de confianza del 95%: 5,3-18,3).

**Conclusión:**

La retención temprana en la atención profiláctica previa a la exposición al VIH fue significativamente mayor entre las trabajadoras del sexo de la República Unida de Tanzania que utilizaron la aplicación *Jichunge* que entre las que no lo hicieron. No obstante, más de dos tercios de las trabajadoras del sexo que utilizaron la aplicación no acudieron a los servicios de seguimiento al cabo de un mes, lo que sugiere que se necesitan intervenciones adicionales.

## ملخص

**الغرض:**

تقييم تأثير تدخل الخدمة الصحية المتنقلة (mHealth) على الاستبقاء المبكر على العاملات في قطاع الجنس، في خدمات العلاج الوقائي قبل التعرض لفيروس نقص المناعة البشرية (HIV) في جمهورية تنزانيا المتحدة.

**الطريقة:**

شملت الدراسة 783 عاملة في قطاع الجنس: 470 عاملة من دار السلام تم منحهن تطبيق *Jichunge * الخاص بخدمة mHealth، بالإضافة إلى العلاج الوقائي القياسي قبل التعرض لفيروس نقص المناعة البشرية (ذراع التدخل)، و313 عاملة من تانغا واللاتي تلقين فقط العلاج الوقائي قبل التعرض للفيروس (ذراع السيطرة). تم اختيار المشاركات عن طريق أخذ العينات من المتقدمات، وتمت متابعتهن لمدة 12 شهرًا. تم تعريف الاستبقاء المبكر على أنه حضور عيادة متابعة العلاج الوقائي قبل التعرض في غضون 28 يومًا من موعد مقرر لمدة شهر واحد بعد بدء العلاج. لتقييم ما إذا كان تطبيق *Jichunge * أدى لمعدل أعلى من الاستبقاء، قمنا بإجراء تحليل النية للعلاج، وتحليل البروتوكول المسبق، باستخدام نموذج التحوّف المعدل من خلال ترجيح الاحتمالات العكسية.

**النتائج:**

تمت ملاحظة الاستبقاء المبكر في رعاية العلاج الوقائي قبل التعرض لفيروس نقص المناعة البشرية في %27.6 (130/470) من المشاركات في ذراع التدخل، و%20.1 (63/313) في ذراع التحكم. في التحليل المعدل للنية للعلاج، تمت ملاحظة الاستبقاء المبكر في %29.4 في ذراع التدخل، و%17.7 في ذراع التحكم (اختلاف المخاطر: 11.8 نقاط النسبة المئوية؛ بفاصل ثقة مقداره %95: 5.3 إلى 18.3).

**الاستنتاج:**

إن الاستبقاء المبكر في العلاج الوقائي قبل التعرض لفيروس نقص المناعة البشرية، كان أكبر بشكل ملموس بين العاملات في القطاع الجنسي في جمهورية تنزانيا المتحدة اللاتي استخدمن تطبيق *Jichunge * أكثر من أولئك اللاتي لم يفعلن ذلك. وعلى الرغم من ذلك، فإن أكثر من ثلثي العاملات في القطاع الجنسي اللاتي يستخدمن التطبيق لم يحضرن خدمات المتابعة بعد شهر واحد، مما يشير إلى أن هناك حاجة إلى تدخلات إضافية.

## 摘要

**目的:**

评估在坦桑尼亚联合共和国针对女性性工作者的人类免疫缺陷病毒 (HIV) 暴露前预防服务中,移动医疗 (mHealth) 干预措施对早期留存率的影响。

**方法:**

该研究纳入了 783 名女性性工作者:其中来自达累斯萨拉姆的 470 名参与者除了接受标准的 HIV 暴露前预防外,还使用了“Jichunge”移动医疗应用软件(应用程序)(即干预组),来自坦噶的 313 名参与者仅接受了暴露前预防(即对照组)。我们采用同伴推动抽样的方法招募参与者,并对其进行了为期 12 个月的随访。早期留存是指在开始治疗 1 个月后预约 28 天内到接受暴露前预防的诊所进行回诊的情况。为了评估“Jichunge”应用程序能否提高留存率,我们使用通过逆概率加权法调整后的回归模型进行了意向性治疗分析和完成治疗分析。

**结果:**

研究发现,干预组中 27.6% (130/470) 的参与者和对照组中 20.1% (63/313) 的参与者继续接受了早期 HIV 暴露前预防性治疗。在调整后的意向性治疗分析,干预组和对照组的早期留存率分别为 29.4% 和 17.7%(风险差异:11.8 个百分点;95% 置信区间:5.3-18.3)。

**结论:**

在坦桑尼亚联合共和国,使用“Jichunge”应用程序的女性性工作者继续接受早期 HIV 暴露前预防性治疗的比率明显高于未使用该应用程序的女性性工作者。尽管如此,在使用该应用程序的性工作者中,有超过三分之二的参与者在 1 个月后未进行回诊,这表明需要采取更多干预措施。

## Резюме

**Цель:**

Оценить влияние вмешательства мобильных технологий в здравоохранении (mHealth) на раннее включение женщин, работающих в секс-индустрии, в программу доконтактной профилактики вируса иммунодефицита человека (ВИЧ) в Объединенной Республике Танзания.

**Методы:**

В исследовании приняли участие 783 женщины, работающие в сфере секс-индустрии: 470 женщин из Дар-эс-Салама, которые пользовались приложением *Jichunge* mHealth («приложение») в дополнение к стандартной доконтактной профилактике ВИЧ («группа вмешательства»), и 313 женщин из Танги, которые получали только доконтактную профилактику («контрольная группа»). Набор участниц осуществлялся с помощью выборки, проводимой респондентами, и наблюдение за ними продолжалось в течение 12 месяцев. Под ранним включением в программу понималось контрольное посещение клиники доконтактной профилактики в течение 28 дней после визита, назначенного на 1 месяц после начала приема препаратов. Чтобы оценить, привело ли использование приложения *Jichunge* к повышению уровня включения в программу, был проведен анализ всех рандомизированных участниц и участниц, выполнивших требования протокола, с использованием регрессионной модели, скорректированной путем взвешивания с обратной вероятностью.

**Результаты:**

Раннее включение в программу доконтактной профилактики ВИЧ наблюдалось у 27,6% (130/470) участниц в группе вмешательства и у 20,1% (63/313) в группе контроля. По результатам анализа, в соответствии с результатами рандомизации, раннее включение наблюдалось в 29,4% случаев в группе вмешательства и в 17,7% случаев в группе контроля (разница в рисках: 11,8 процентного пункта; 95%-й ДИ: 5,3–18,3).

**Вывод:**

Среди женщин, работающих в секс-индустрии в Объединенной Республике Танзания, которые пользовались приложением *Jichunge*, раннее включение в программу доконтактной профилактики ВИЧ было значительно выше, чем среди тех, кто этого не делал. Тем не менее более двух третей работниц секс-индустрии, воспользовавшихся приложением, не явились на контрольный визит через 1 месяц, что говорит о необходимости применения дополнительных мер.

## Introduction

Oral pre-exposure prophylaxis against human immunodeficiency virus (HIV) infection is recommended for high-risk groups as part of efforts to end the public health threat of HIV and acquired immune deficiency syndrome (AIDS) by 2030.^1^ Although clinical trials have shown that pre-exposure prophylaxis with antiretroviral medications is highly effective against HIV transmission,^2^^–^^5^ low retention rates and suboptimal adherence to treatment are major obstacles to its effectiveness.^6^^–^^9^ Moreover, retaining female sex workers in pre-exposure prophylaxis services may be particularly challenging because of structural, interpersonal and community factors.^9^^–^^13^ Innovative interventions that can address population-specific barriers to continued use of pre-exposure prophylaxis may have a positive effect among female sex workers and other underserved populations.

In the United Republic of Tanzania, HIV pre-exposure prophylaxis has been provided to population groups with a high risk of infection since 2021.^14^ According to a 2017 biobehavioural survey, the prevalence of HIV infection among female sex workers in the country was approximately 15%, more than twice the prevalence among women in general.^15^^,^^16^ Despite this, the acceptability and uptake of pre-exposure prophylaxis among female sex workers in the United Republic of Tanzania and many other countries were low.^11^^,^^17^^,^^18^ There was, therefore, a need for additional interventions and supportive services, such as better information about pre-exposure prophylaxis, reminders and digital consultations with health workers, that could help address some of the social and structural challenges affecting the implementation of pre-exposure prophylaxis.

Mobile health (mHealth) approaches, which involve providing health care through mobile devices, appear to be effective in promoting health services in various settings.^19^ Moreover, the increasing availability of smartphones and growing internet use in sub-Saharan Africa create an opportunity to exploit smartphone-mediated health interventions on the continent.^20^ In particular, mHealth interventions could help promote the utilization of HIV prevention services by reducing structural barriers.^21^^–^^23^ In the past, however, HIV prevention-related mHealth services have focused mainly on men who have sex with men, primarily in high-income countries.^24^^–^^26^ As a result, little evidence is available about the effectiveness of these interventions in other important population groups or in key population groups in sub-Saharan Africa. 

Guided by different theoretical models, such as information–motivation–behavioural skills models and the behaviour change model for internet interventions,^27^^,^^28^ a smartphone-based mHealth application (app), called *Jichunge*, was developed as part of a study of HIV pre-exposure prophylaxis users in the United Republic of Tanzania. The study involved participants from two key population groups: female sex workers and men who have sex with men. The aim of our substudy was to evaluate the effect of the *Jichunge* app on the early retention of female sex workers in pre-exposure prophylaxis services.

## Methods

The study was conducted in two cities in the United Republic of Tanzania: Dar es Salaam (intervention arm) and Tanga (control arm). According to the 2016–2017 *Tanzania HIV impact survey*,^16^ the weighted prevalence of HIV infection among females aged 15 years and older was 6.2% in Tanga and 6.8% in Dar es Salaam. In both cities, the roll-out of HIV pre-exposure prophylaxis was in an early phase during the study. Although they have similarities, the two cities are different in, for example, population size and economic profile.

### Study design

The study was quasi-experimental and formed part of a pragmatic trial of HIV pre-exposure prophylaxis roll-out in the United Republic of Tanzania (PREPTA) involving female sex workers and men who have sex with men, which has been previously described.^29^^,^^30^ These two target groups were followed for 12 months to assess the use of pre-exposure prophylaxis and other related variables.

In the intervention arm, study participants downloaded for free the *Jichung*e mobile health app, which was designed to promote and support the use of HIV pre-exposure prophylaxis and has been described in previous publications.^30^^,^^31^ The app featured: (i) educational information about pre-exposure prophylaxis and HIV; (ii) free online consultations with a doctor or peer educator; (iii) gamification to promote regular pill-taking and motivate engagement with the app; (iv) an online forum where participants could chat with other pre-exposure prophylaxis users; and (v) customized reminders about daily pill-taking.

In the control arm, participants received standard HIV pre-exposure prophylaxis as per national guidelines.^14^ This care included being provided with all antiretroviral medications and undergoing all required tests free of charge, as well as receiving in-person counselling during an initial clinical consultation and having regular follow-up consultations about treatment. Peer educators, working with clinics and nongovernmental organizations responsible for implementing pre-exposure prophylaxis, called participants to encourage them to attend scheduled follow-up visits.

### Participants

The study involved female sex workers who: (i) were starting HIV pre-exposure prophylaxis in either Dar es Salaam or Tanga; (ii) were at least 18 years of age; (iii) had received payment for sex in the past 3 months; and (iv) had lived in the study sites for the previous 6 months. In addition, participants in the intervention arm had to own a smartphone at recruitment. Women who did not give consent were excluded.

We estimated the required sample size using the formula for comparing proportions in two groups.^32^^,^^33^ As no data on the retention of female sex workers in HIV pre-exposure prophylaxis services were available when the study was designed, we used a retention rate of 50% as the baseline estimate. A proportion of 50% has been shown to result in an optimal sample size when the true portion is expected to lie between 10% and 90%.^34^ In estimating the number of participants needed to detect a difference of 15 percentage points in the retention rate in pre-exposure prophylaxis services between the intervention and control arms, we set a confidence level of 95%, a margin of error of 5% and a statistical power of 80%. These criteria resulted in a sample size of 422 participants (211 in each study arm). Then, on applying a design effect of two (i.e. def = 2) to minimize selection bias due to non-random sampling, we obtained a minimum sample size of 676 participants (338 in each study arm). After adjusting for a potential loss to follow-up of 20%, the final sample size was 846 participants (423 in each study arm).

### Study procedures

#### Recruitment

Participants were recruited using respondent-driven sampling between March and June 2021 in Dar es Salaam, and between February and April 2022 in Tanga. Respondent-driven sampling is used in population groups for which there is no existing sampling frame.^33^^,^^35^ This chain, referral and sampling approach starts with the selection of a few initial participants, referred to as seeds. In this study, peer educators and researchers identified the seeds. Each seed was given coupons to recruit their peers into the study – this step produced wave 1 of study participants. In turn, these individuals were given coupons to recruit additional participants. For each study arm, we started by recruiting three seeds with a range of ages, places of residence, educational levels, types of sex work and socioeconomic status. Subsequently, we continuously assessed the characteristics of participants being recruited, and added more seeds to maximize the diversity of the sample and to speed up recruitment when necessary. Overall, we recruited nine seeds in Dar es Salaam and five in Tanga.

#### Screening for eligibility

Trained peers screened participants for study eligibility. Subsequently, health workers screened for eligibility for pre-exposure prophylaxis, in accordance with the national framework.^14^ Eligibility for pre-exposure prophylaxis included: (i) a negative test result for HIV infection; (ii) no sign of acute HIV infection; (iii) a serum creatinine level above 60 μmol/L; and (iv) willingness to start pre-exposure prophylaxis. Eligible participants were provided with medications for 30 days before being invited to take part in the study. They were then asked for written consent. In addition, participants in the intervention arm attended an introductory session on the *Jichunge* app before being interviewed.

#### Data collection

At baseline, we collected information on the participants’ sociodemographic characteristics, the type of sex work they engaged in, and other social structural factors using a questionnaire administered during face-to-face interviews with trained research assistants. Participants were given an appointment for a pre-exposure prophylaxis follow-up visit 1 month after starting treatment, during which they received a further supply of medication. They were free to choose whether or not to accept a pre-exposure prophylaxis prescription during the visit. We contacted participants by telephone and encouraged them to attend the clinic even if they did not use pre-exposure prophylaxis or the *Jichunge* app. We recorded the date of the visit to assess retention in pre-exposure prophylaxis care after 1 month. All information was collected using hand-held tablets linked to a secure server for data storage and processing (Services for Sensitive Data, or TSD, University of Oslo, Oslo, Norway).^36^

From the app, we were able to continuously collect data on participants’ use of its different functions (i.e. opening the app, registering taking medicine, reading editorial content, accessing online consultations and entering the online discussion forum), whether or not they attended follow-up visits. We extracted data on clinic visits from participants’ clinic files by a trained health worker using a short form and included: (i) the date of the clinic visit; (ii) the tests performed; (iii) pre-exposure prophylaxis usage; (iv) the date of next appointment; and (v) the number of pills dispensed.

#### Payment

After the face-to-face interview, each participant was paid a modest amount (8000 Tanzanian shillings, about 3.5 United States dollars, US$) as compensation for transport costs and time spent at the study site. In addition, each participant received 4000 Tanzanian shillings (about US$ 1.7) for each peer they referred to the study via the respondent-driven sampling protocol.

### Variables

The outcome variable was early retention in HIV pre-exposure prophylaxis care, which was defined as attending a pre-exposure prophylaxis follow-up clinic within 28 days of a scheduled appointment. This definition is in line with previous definitions of early retention in pre-exposure prophylaxis programmes.^37^^,^^38^ Early retention is critical because complications and challenges experienced early are highly likely to have an effect on the long-term use of pre-exposure prophylaxis.^39^ The main exposure of interest was receiving the *Jichunge* app.

#### Potential confounders

Due to the quasi-experimental design, systematic differences may have existed between participants in the intervention and control arms. We identified potential confounders among sociodemographic characteristics, sex work characteristics, and structural factors that were associated with retention in pre-exposure prophylaxis care and that could have differed between the arms. Potential confounders included: (i) age; (ii) marital status; (iii) educational level; (iv) condom use with the last paying client; (v) agreeing to sex without a condom for an increased payment; (vi) financial difficulties due to health-care spending; (vii) knowledge of pre-exposure prophylaxis; (viii) high self-perceived risk of HIV infection; (ix) social support; (x) perceived pre-exposure prophylaxis stigma; (xi) perceived sex work stigma; and (xii) pre-exposure prophylaxis behavioural skills (i.e. self-efficacy).

We measured scale variables as described in our previous publications.^29^^–^^31^^,^^40^ We assessed knowledge of pre-exposure prophylaxis at baseline using eight true or false questions, with participants who answered more than six questions correctly being categorized as highly knowledgeable. We assessed social support using an 8-item Likert scale adapted from the Duke–University of North Carolina (UNC) Functional Social Support Questionnaire.^41^ For each item, participants were asked to choose one of five responses: 1. Much less than I would like; 2. Less than I would like; 3. Some, but would like more; 4. Almost as much as I like; or 5. As much as I like. We computed the total score for all items and considered a total score below 32 as indicating inadequate social support. The social support scale had a Cronbach’s *α* of 0.88, which signified high reliability.

Perceived sex work stigma and pre-exposure prophylaxis stigma were assessed using 13 and 10 scale items, respectively. Each item had five response options: 1. Strongly disagree; 2. Disagree; 3. Neither disagree nor agree; 4. Agree; and 5. Strongly agree. A total score was computed from responses on all items. Thereafter, perceived sex work stigma was categorized as: (i) low for a score of 26 or less; (ii) moderate for a score between 27 and 38; or (iii) high for a score of 39 or above. For perceived pre-exposure prophylaxis stigma, a score above 30 was considered high. Cronbach’s *α* for the two stigma measures was 0.84 and 0.88, respectively. 

We assessed pre-exposure prophylaxis behavioural skills using six questions adapted from the information–motivation–behavioural skills model, which has been validated among high-risk drug users.^42^ Participants rated their confidence in pre-exposure prophylaxis in various situations, such as incorporating pre-exposure prophylaxis into their daily routine and attending clinic appointments. Responses were recorded on a five-point scale, ranging from 1 for “Not at all confident” to 5 for “Very confident.” A total score above 24 indicated high pre-exposure prophylaxis behavioural skills. Cronbach’s *α* for the behavioural skills scale was 0.77.

### Statistical analyses

We used propensity scores estimated from a binary logistic regression model to weight the data using stabilized inverse probability weights. The propensity score approach is often used for analysing non-randomized experiments and observational studies because of its powerful ability to balance treatment groups influenced by a large number of confounders. We used this approach primarily to ensure that baseline confounders were balanced before estimating the effect of the intervention. In total, 17 variables identified as potential confounders were included in the propensity score model to estimate inverse probability weights. Probability weights balance the distribution of baseline characteristics between intervention and control arms, thereby enabling unbiased estimates of a treatment effect to be obtained in non-randomized studies.^43^^,^^44^ We assessed the balance of baseline covariates before and after weighting the standardized mean differences between study arms. A standardized mean difference less than 10% was considered balanced.^43^^,^^45^

Finally, we estimated the average treatment effect by calculating the difference in the rate of retention in HIV pre-exposure prophylaxis care between the intervention and control arms. In addition, we estimated a risk ratio using a generalized log–binomial regression model adjusted for inverse probability weights. Both intention-to-treat and per-protocol analyses were used to estimate the effect of the *Jichunge* app. The intention-to-treat analysis included all participants regardless of their use of the app. In the per-protocol analysis, we compared retention in pre-exposure prophylaxis services between participants who were using the app after 1 month and all participants in the control arm. All analyses were performed using Stata v. 18 (StataCorp LLC, College Station, United States of America).

### Ethical approval

The study was approved by the Muhimbili University of Health and Allied Sciences ethics review committee. In addition, as part of the PREPTA trial, the study was also approved by the National Health Research Ethics Committee in the United Republic of Tanzania and by the Regional Committee for Medical and Health Research in Norway. All participants received information about the study and provided written consent before enrolment. All research activities were conducted in accordance with the Declaration of Helsinki as highlighted in the guidelines and ethical regulations of the United Republic of Tanzania and Norway. The study was registered with the Pan African Clinical Trials Registry (PACTR202003823226570).^46^

## Results

We recruited 783 participants: 470 in the intervention arm and 313 in the control arm. At baseline, their mean age was 27.8 years (standard deviation, SD: 6.2) in the control arm and 26.4 years (SD: 5.6) in the intervention arm. Overall, 58.9% (277/470) of participants in the intervention arm had had secondary or higher education compared with 45.7% (143/313) in the control arm (Table 1). In addition, 55.6% (174/313) in the control arm reported using a condom the last time they had sex with a client, and 62.6% (196/313) reported agreeing to sex without a condom for an increased payment. Around 71% of participants in both study arms regarded themselves as being at a high risk of acquiring an HIV infection. After weighting the data, the distribution of baseline covariates was balanced in the intervention and control arms (Table 1).

**Table 1:** Baseline characteristics of female sex workers, study of an mHealth intervention’s effect on retention in HIV pre-exposure prophylaxis care, United Republic of Tanzania, 2021–2022

Variable	No. participants (%)^a^				
	Unweighted data			Weighted data^b^	
	Control arm (*n* = 313)	Intervention arm (*n* = 470)		Control arm (*n* = 313)	Intervention arm (*n* = 470)
Age in years, mean (SD)	27.8 (6.2)	26.4 (5.6)		26.7 (6.0)	26.9 (5.6)
Received secondary or higher education	143 (45.7)	277 (58.9)		172 (54.9)	247 (52.9)
Never married	221 (70.6)	361 (76.8)		236 (75.4)	346 (73.6)
Living with husband, boyfriend or family	238 (76.0)	305 (64.9)		217 (69.3)	325 (69.1)
Had given birth	272 (86.9)	327 (69.6)		234 (74.8)	360 (76.6)
No income source other than sex work	164 (52.4)	283 (60.2)		183 (58.5)	269 (57.2)
Has steady partner	124 (39.6)	305 (64.9)		166 (53.0)	253 (53.8)
Used a condom with the last client	174 (55.6)	215 (45.7)		145 (46.3)	229 (48.7)
Agrees to sex without a condom for a higher payment	196 (62.6)	249 (53.0)		172 (55.0)	270 (57.4)
Experiencing financial difficulties due to health-care spending	127 (40.6)	244 (51.9)		155 (49.5)	224 (47.7)
Experienced physical violence in the past 12 months	123 (39.3)	180 (38.3)		108 (34.5)	183 (38.9)
Perceived high risk of HIV infection	223 (71.2)	334 (71.1)		232 (74.1)	337 (71.7)
Highly knowledgeable about pre-exposure prophylaxis	124 (39.6)	232 (49.4)		136 (43.5)	215 (45.7)
Inadequate social support	144 (46.0)	283 (60.2)		176 (56.2)	267 (56.8)
Perceived sex work stigma score,^c^ mean (SD)	32.3 (4.0)	30.9 (7.0)		32.0 (3.7)	31.7 (7.2)
Perceived pre-exposure prophylaxis stigma score,^d^ mean (SD)	25.3 (7.3)	25.4 (7.5)		25.2 (7.7)	25.5 (7.6)
Pre-exposure prophylaxis behavioural skills score,^e^ mean (SD)	27.8 (2.9)	26.8 (3.3)		27.2 (3.3)	27.2 (3.1)

HIV: human immunodeficiency virus; SD: standard deviation.

^a^ All values are for the number and percentage of female sex workers, unless otherwise stated.

^b^ Data were weighted using stabilized inverse probability weights based on propensity scores estimated from a binary logistic regression model.

^c^ Perceived sex work stigma was low for a score ≤ 26, moderate for a score of 27–38 and high for a score ≥ 39.

^d^ Perceived pre-exposure prophylaxis stigma was high for a score > 30.

^e^ Pre-exposure prophylaxis behavioural skills were high for a score > 24.

### Use of *Jichunge* app

We have reported detailed information on the use of the app in a previous publication.^30^ In brief, 74.0% (348/470) of participants in the intervention arm were using the app after 1 month. With regard to specific app functions, 71.7% (337/470) had registered pill-taking; 47.0% (221/470) had consulted editorial content; 34.3% (161/470) had engaged in discussions with other pre-exposure prophylaxis users; and 20.6% (97/470) had consulted a doctor or peer educator.

### Retention in pre-exposure prophylaxis services

Of the 783 study participants, 193 (24.7%) were retained in HIV pre-exposure prophylaxis care after 1 month. In the intervention arm, 27.6% (130/470) were retained, compared with 20.1% (63/313) in the control arm.

Table 2 shows the crude and adjusted findings of the intention-to-treat analysis (470 participants in the intervention arm and 313 in the control arm) and the per-protocol analysis (348 in the intervention arm who used the *Jichunge* app and 313 in the control arm). In the crude analysis, the estimated rate of retention in pre-exposure prophylaxis care after 1 month (allowing for a delay of up to 28 days) was 27.6% (95% confidence interval; CI: 23.6–31.7) in the intervention arm and 20.1% (95% CI: 15.7–24.6) in the control arm. The risk difference was 7.5 percentage points (95% CI: 2.5–13.5). After adjusting for inverse probability weights, the risk difference attributable to the *Jichunge* app was 11.8 percentage points (95% CI: 5.3–18.3). In the per-protocol analysis, the risk difference attributable to the app was 17.8 percentage points (95% CI: 10.3–25.2).

**Table 2:** Effect of *Jichunge* mHealth intervention on early retention^a^ in HIV pre-exposure prophylaxis services, United Republic of Tanzania, 2021–2022

Measure	Estimate (95% CI)	*P*
**Intention-to-treat analysis (unadjusted)**		
Retention rate in intervention arm, %	27.6 (23.6–31.7)	NA
Retention rate in control arm, %	20.1 (15.7–24.6)	NA
Difference in retention rate between intervention and control arms, percentage points	7.5 (2.5–13.5)	0.016
Risk ratio	1.37 (1.05–1.79)	0.019
**Intention-to-treat analysis (adjusted)^b^**		
Retention rate in intervention arm, %	29.4 (24.8–34.1)	NA
Retention rate in control arm, %	17.7 (13.2–22.3)	NA
Difference in retention rate between intervention and control arms, percentage points	11.8 (5.3–18.3)	< 0.001
Risk ratio	1.67 (1.23–2.28)	0.001
**Per-protocol analysis (adjusted)^b^**		
Retention rate in intervention arm, %	36 (30.1–42)	NA
Retention rate in control arm, %	18.3 (13.8–22.8)	NA
Difference in retention rate between intervention and control arms, percentage points	17.8 (10.3–25.2)	< 0.001
Risk ratio	1.97 (1.45–2.69)	< 0.001

CI: confidence interval; HIV: human immunodeficiency virus; NA: not applicable.

^a^ Early retention was defined as attending a follow-up clinic within 28 days of an appointment scheduled for 1 month after the start of pre-exposure prophylaxis.

^b^ Adjusted by inverse probability weighting.

## Discussion

In both intention-to-treat and per-protocol analyses, we found that the rate of early retention (i.e. at 1 month) in pre-exposure prophylaxis care was significantly higher in participants who received the *Jichunge* mHealth intervention than in those who did not. However, even with the intervention, the overall retention rate was low.

Low retention rates have been reported in Kenya, where only 24% (212/899) of women at an increased risk for HIV infection were retained in a pre-exposure prophylaxis programme at 3 months,^47^ and in Gauteng Province, South Africa, where 27% of 1307 young female sex workers attended a 1-month pre-exposure prophylaxis follow-up.^8^ In contrast, the retention rate was 55% among 427 young female sex workers in an HIV prevention trial conducted in Cape Town and Johannesburg, South Africa, and Harare, Zimbabwe.^48^ Nevertheless, even this higher retention rate is suboptimal. Consequently, there is a need for innovative strategies to increase retention among female sex workers in sub-Saharan Africa, which may include, but not be limited to, mHealth interventions.

Our study demonstrates that an mHealth intervention can increase retention in pre-exposure prophylaxis care in a real-world setting, at least to some extent. Similar findings were reported from Kenya, where two-way text messaging increased attendance at a first HIV pre-exposure prophylaxis follow-up visit among high-risk women.^49^ Studies among men who have sex with men and other key populations in the United States have reported similar findings.^24^^,^^25^^,^^50^ Notably, the effectiveness of mHealth interventions in enhancing engagement with pre-exposure prophylaxis can vary. A study conducted in New York, United States, found that an mHealth intervention used did not increase adherence to pre-exposure prophylaxis among either men who have sex with men or transgender women.^51^ Another study in Thailand reported that mHealth support did not increase adherence compared with youth-friendly services alone.^52^ These discrepancies may arise from differences in app design or functionality, the target population or environmental factors, which highlights the need for tailored approaches involving the design of the mHealth intervention, the characteristics of the user and the mechanisms of behavioural change.^28^

The cost of the *Jichunge* mHealth intervention requires some consideration. On the provider side, the main costs were related to the people who developed and operated the *Jichunge* app, who, for example, dealt with technical issues, developed editorial content and provided online consultations. On the user’s side, there were the costs of owning a smartphone and having internet connectivity. Although we did not conduct a cost–benefit analysis, we believe the benefits would outweigh the costs, but this needs to be studied.

Our study has strengths and limitations. One strength is that it was a pragmatic trial that provided evidence about the effectiveness of an mHealth intervention in a real-world setting. Thus, its findings provide valuable additional insights into the implementation science of mHealth interventions for preventing HIV infection among key population groups in sub-Saharan Africa and beyond. A second strength is the large sample size, which ensured sufficient statistical power and stable estimates. A third strength is that we both performed intention-to-treat and per-protocol analyses, thereby providing information on what would happen in the optimal situation where all users engaged with the *Jichunge* mHealth intervention.

There are also important limitations. First, we evaluated only the effect of the *Jichunge* mHealth intervention on retention in HIV pre-exposure prophylaxis care, which is just one of three cascade steps in the use of pre-exposure prophylaxis. Future studies could evaluate the effect of mHealth interventions on the uptake of, and adherence to, pre-exposure prophylaxis. Second, our propensity score model considered only observable individual and interpersonal baseline confounders. We did not assess the influence of community or other organizational factors on retention in pre-exposure prophylaxis care in the two study cities. Consequently, residual, unmeasured confounding was possible.

In summary, the substantial support provided by the *Jichunge* app increased early retention in HIV pre-exposure prophylaxis care among female sex workers in Dar es Salaam. However, more than two thirds did not continue to engage with services after 1 month, which underscores the need for supplementary interventions. Nevertheless, our findings suggest that smartphone-based mHealth interventions hold the potential to promote retention in pre-exposure prophylaxis care among population groups at risk of HIV infection in East Africa and beyond.
